# Education and Health: A Care Bond. School-Nursing Model for Colombia

**DOI:** 10.17533/udea.iee.v38n2e05

**Published:** 2020-07-10

**Authors:** María Clemencia Ferro Carvajalino, Andrea Fuentes Ramírez, Tania Catalina Chinchilla Salcedo, Beatriz Sánchez Herrera

**Affiliations:** 1 Nurse, Specialist. Nurse of the Escuela Marymount, Bogotá, Colombia. Email: clema64@yahoo.com Escuela Marymount Bogotá Colombia clema64@yahoo.com; 2 Nurse, Specialist. Nurse of the Escuela de Campoalegre, Sopó, Colombia. Email: andreafr2424@yahoo.com Escuela de Campoalegre Sopó Colombia andreafr2424@yahoo.com; 3 Nurse, Masters. Professor, Universidad de La Sabana, Chía, Colombia. Email: Tania.chinchilla@unisabana.edu.co Universidad de la Sabana Universidad de La Sabana Chía Colombia Tania.chinchilla@unisabana.edu.co; 4 Nurse, Masters. Professor, Universidad de La Sabana Chía, Colombia. Email: clara.sanchez@unisabana.edu.co Universidad de la Sabana Universidad de La Sabana Chía Colombia clara.sanchez@unisabana.edu.co

**Keywords:** school nursing, nursing methodology research, models, nursing, nursing theory., de enfermería escolar, investigación metodológica en enfermería, modelos de enfermería, teoría de enfermería., serviços de enfermagem escolar, pesquisa metodológica em enfermagem, teoria de enfermagem.

## Abstract

**Objective.:**

To describe the construction and validation process of a *Model of professional practice of school nursing for Colombia*.

**Methods.:**

Study under the approach of “methodological research in nursing” carried out by the Colombian network of school nursing, with the participation of 26 nurses from different institutions in a research developed in three stages: revision of antecedents, identification and prioritizing of assumptions to construct the model, and validation of the preliminary proposal with the participants and with a group of experts.

**Results.:**

The study presents the components that were part of the construction of the *model of professional practice of school nursing for Colombia*, which includes the four meta-paradigmatic elements of this professional discipline: the receptor of care, the context, nursing, and health, as well as the prioritized assumptions that indicate how these elements interact in achieving the student´s wellbeing and that of the education community. It includes the report from a focal validation group with the participants in which they summarize as *education and health: a care bond*, and the concept by experts on such.

**Conclusion.:**

The *Model of professional practice of school nursing for Colombia: education and health: a care bond*, constructed in participative manner with nurses experts in the field and validated with theoretical experts complies with the international guides for the design of this type of theoretical construction and permits guiding the care goals of students, maintain the autonomy of the nurses and their interprofessional participation in this field.

## Resumen

**Objetivo.:**

. Describir el proceso de construcción y validación de un *Modelo de práctica profesional de enfermería escolar para Colombia*.

**Métodos.:**

Estudio bajo el abordaje de “investigación metodológica en enfermería” realizado por la Red Colombiana de Enfermería Escolar. Participaron 26 enfermeras de diferentes instituciones en una investigación desarrollada en tres etapas: revisión de antecedentes, identificación y priorización de supuestos para la construcción del modelo, y validación de la propuesta preliminar con los participantes y con un grupo de expertos.

**Resultados.:**

. Se presentan los componentes que hicieron parte de la construcción del *Modelo de práctica profesional de enfermería escolar para Colombia*, que incluyen los cuatro elementos meta-paradigmáticos de esta disciplina profesional: el receptor del cuidado, el contexto, la enfermería y la salud, así como los supuestos priorizados, que indican la forma en que estos elementos interactúan en el logro del bienestar del escolar y de la comunidad educativa. Se incluye el reporte de un grupo focal de validación con los participantes en el que lo resumen como *educación y salud un vínculo de cuidado*, y el concepto de expertos sobre el mismo.

**Conclusión.:**

. El *Modelo de práctica profesional de enfermería escolar para Colombia: educación y salud un vínculo de cuidado*, construido de forma participativa con enfermeras expertas en el campo y validado también con expertos teóricos cumple con las guías internacionales para el diseño de este tipo de construcción teórica y permite orientar las metas del cuidado del escolar, mantener la autonomía de las enfermeras y su participación interprofesional en este campo.

## Resumo

**Objetivo.:**

. Descrever o processo de construção e validação de um *Modelo de estágio profissional de enfermagem escolar para Colômbia*.

**Métodos.:**

Estudo sob a abordagem de “investigação metodológica em enfermagem” realizado pela *Rede Colombiana de Enfermagem Escolar*. Participaram 26 enfermeiras de diferentes instituições em uma investigação desenvolvida em três etapas: revisão de antecedentes, identificação e priorização de supostos para a construção do modelo, e validação da proposta preliminar com os participantes e com um grupo de especialistas.

**Resultados.:**

. Se apresentam os componentes que fizeram parte da construção do Modelo *de estágio profissional de enfermagem escolar para Colômbia*, que incluíram os quatro elementos metaparadigmáticos desta disciplina profissional: o receptor do cuidado, o contexto, a enfermagem e a saúde, assim como os supostos priorizados, que indicam a forma em que estes elementos interagem na conquista do bem-estar do escolar e da comunidade educativa. Se inclui o relatório de um grupo focal de validação com os participantes no qual o resumo como *educação e saúde um vínculo de cuidado*, e o conceito de especialistas sobre o mesmo.

**Conclusão.:**

. O *Modelo de estágio profissional de enfermagem escolar para Colômbia: educação e saúde um vínculo de cuidado*, construído de forma participativa com enfermeiras especialistas no campo e validado também com especialistas teóricos que cumpre com os guias internacionais para o desenho deste tipo de construção teórica e permite orientar as metas do cuidado do escolar, manter a autonomia das enfermeiras e sua participação interprofissional neste campo.

## Introduction

Promoting childhood health is undoubtedly a strategy that impacts upon the wellbeing and progress of the people.(1) The World Health Organization indicates, in said sense, that health promotion schools seeking to propitiate favourable conditions for children and adolescents to have a better present and future quality of life are a priority.(2) The professional nursing discipline has had important progress from its theoretical development, especially the broad-range conceptual models;(3) however, although progress is known in the environment with models of professional practice,(4) none was found reported in the school nursing field. Nevertheless, it has been documented that the presence of school nurses generates a positive difference in care and in the health of the school population;(5) and the performance of their role has been described.(6)

In Latin America, school nursing originated as a response to need to provide quality care to children.(7) However, it has been indicated that the support based on evidence to back the presence of nursing in schools continues being weak.(8) In Colombia, school nurse works have been carried out in private schools that seek to improve the practice permanently: this is how in 2007 and until 2011, the Colombian Association of School Nursing (ACENES, for the term in Spanish) was organized, which was transformed into the Committee of School Nursing of the National Association of Nurses of Colombia (CEE-ANEC, for the term in Spanish) between 2015 and 2018. In June 2018, the very same group decided to start the work of the Colombian Network of School Nursing, together with Universidad de la Sabana, being constituted in 2019 to qualify nursing care provided to students and other members of the education community, for its promotion and protection, prevention of health problems in the school environment and recovery and rehabilitation of health through an integral, interdisciplinary, and inter-sector approach.(9,10) In continuing with this work, the model of professional practice of school nursing for Colombia was developed, whose construction and validation process was the objective of the present work.

## Methods

This was a study conducted with the approach of methodological research in nursing,(11) within the framework of the Colombian Network of School Nursing, carried out in three stages: (i) *Revision of national antecedents*, which analysed the nursing practice through a series of individual interviews and secondary sources of information that showed the need to have a school-nursing model; (ii) *Interviews to 26 school nurses*, identifying and prioritizing the basic aspects of the school-nursing model which, from three guides developed by the Faculty of Nursing and Rehabilitation at Universidad de la Sabana, se identified initially the four meta-paradigmatic concepts in relation with the nursing practice in this field. Then, the assumptions of school nursing were specified with this same group, indicating how the concepts described should be articulated in practice to reflect the desired scenarios. Upon defining the assumptions, these were classified under parameters of importance and governance for school nursing through the iGo matrix.(12) In this matrix, participants scored from 0 to 500 each condition and for the result value of these scores was averaged, as high, medium, or low, according to parameters from the same methodology where, from the average and extreme values, the distribution of the ranges arises. This was how from the analysis of prioritized components, emerged the initial version of the model. For validation, a focal group was carried out with the participants who were asked to indicate if the model proposed reflected their contributions, knowledge, and experience. From the suggestions received, a final adjustment was made and a name, a slogan, was sought and a summary of the fundamental was made to communicate it with ease; (iii) Upon agreeing on the definitive model with the participating group, *the process of construction and components* was revised with support from two recognized experts in the field of theoretical construction of nursing, to verify its level of internal coherence and the degree of compliance with the international criteria in effect for this type of theoretical development.(13)

The study was conducted within the Colombian Network of School Nursing and received institutional approval from Universidad de la Sabana, within the project “Validation of a Strategy to Improve the Institutional Nursing Practice in Teaching-Care Alliance”. All the participants signed a written informed consent. 

## Results

From the theoretical review, the group of participating expert nurses was defined, along with an account of their prior accomplishments and the state of the art in this field, which generated comparison parameters of the present results, as presented at the end of this section in the Discussion. In the second stage, the components of the model of *professional practice of school nursing for Colombia* emerged*,* which include the concepts, theoretical assumptions, and their prioritizing in the following order:

### The four meta-paradigmatic concepts

First, concept of person

The subject of care by school nursing. The subjects of care by a school nurse are children or adolescents and with them the whole education community. School-age children or adolescents, registered in a school as students, are for the nurse unique persons. The school nurse also cares for the teaching staff, including directors, administrative personnel and those in general services, and sometimes their children or relatives, who could be present in the school receiving services of wellbeing. Through this bond with the children or in special events, care is provided to their parents, guardians, students from other schools and to the members of their families who attend different activities (academic, cultural, sports, or recreational) or to whom - for different reasons - are in the school facilities or accompanying activities under the responsibility of the institution as suppliers, contractors, or visitors. Care by the school nurse encompasses other colleagues who when exercising common tasks need accompaniment, socialization of their activity or ratification of their work.

Second, concept of health

School nursing seeks to contribute to the education of happy, sensitive, critical, and authentic individuals capable of constructing freely their own history and be able to contribute creatively to the cultural, socioeconomic, and environmental environment, creating a conscience of responsibility and respect for the community and for themselves through continuous renovation. The school nurse seeks to respond to the different health needs and situations presented by the students and other members of the education community looking for their wellbeing and guaranteeing their safety. For the school nurse, it is a priority to propitiate the inclusion of children or adolescents, facilitating all to develop their school activities. The nurse looks for the children or adolescents and the rest of the school community to become stronger in the capacity for caring and for them to make decisions in favour of a healthy life. 

Third, concept of environment

The context of caring by school nursing. The context of caring for children and adolescents is, in the first instance, the school and bears in mind the rest of the environments where the children and adolescents develop their daily lives or those that can affect their wellbeing. The school context, according with the institution’s mission, has education as its purpose. This context is flexible and includes, among others, the classrooms, the cafeteria, recreational spaces, and transportation; keeping in mind extracurricular activities, pedagogical outings, and local, national and international excursions. The school environment permits strengthening a culture of health care, but it also has risks that can affect it and which must be understood and minimized. This care context includes the families, directors, teachers, and other members of the school community. The school, at internal level, governs relationships through a school life manual, according to current regulations and, at the external level, is related with other schools for which it can be local, national, and international referent. Although the schools operate amid of a globalized and changing world, they cannot forget their responsibilities of preparing students with ethics and values in response to the institution’s philosophy. Both the nurse and nursing are part of the school context, within this setting, nurses manage information, equipment, and special supplies related with health care and must guarantee the privacy and confidentiality of the information and articulate care with the required interdisciplinary, interinstitutional, and intersectoral support. Health care provided to the school community is governed by regulations from the educational, health, and work systems. 

Fourth, concept of nursing; the role of nursing for the school nurse

School nurses carry out their practice through the exercise of different roles, which include: 

A *caring* role, where the nurse cares during the school or extracurricular day, bearing in mind the age, condition, support network of the person being cared for, and the institutional values of respect, responsibility, solidarity, as well as of cordiality with good interpersonal relationships characterized by an environment of trust, which requires empathy, discretion, and confidentiality in the management of information. For such, it is important to always recognize the child or adolescent who requires their service as a unique person; the nurse needs to look in their eyes, listen to them, validate what they express, and care for their ailment or request. The school nurses guide their care with a care plan denominated nursing care plan, which includes: assessment, diagnosis of priorities, planning, intervention, follow up, and evaluation. Said plan must be individualized contextualized, respond to special conditions, and guarantee safety and continuity in care, as well as in the administration of supervised treatments and enhancement of individual skills, amid a process of the development of the child or adolescent. 

The nurse cares for the person, under professional criterion and following the pertinent guidelines in each case. For this, the nurse seeks support on the best evidence available and documents said care. Nurses care for acute or emergency situations, guaranteeing the assessment, first aid, and remission, according to the case, looking to stabilize and protect the person, until referral to the adequate instance with the required company, understanding and prioritizing the family’s importance in this situation. 

Nurses *advocate* when protecting the school community, especially children and adolescents as vulnerable individuals who require care actions, until their parents and/or guardians arrive. They *advise* on health care to students, coworkers, families, and other members of the school community to clear doubts related with health and strengthen self-care practices. They also advise those attending the context and health to generate healthy environments.

Nurses *lead healthy* behaviour in school, where they administer care guided under parameters standardized through guides or protocols. Within their action with the school community, they coordinate their activities with children and adolescents, directors, professors, parents, health professionals, and those responsible for services. Moreover, nurses identify risks, elaborate emergency plans and support management of workplace safety and health in coordination with pertinent instances to guarantee the highest possible level of wellbeing.

The *managerial* function implies for them the development and implementation of indicators related with the nursing service, research, and analysis of health trends to provide pertinent recommendations and make decisions according to the priorities of the school community, responding to health problems that affect it or may come to affect it. 

Nurses, as *collaborators*, participate as members and representatives of the school community and of the institution in different committees and orientation activities related with the student’s wellbeing and health, including the Institutional Educational Project (PEI, the term is in Spanish), the School Life Manual, and the pedagogical projects. They also participate as *builders and evaluators of policies* of school health, seeking for these to have the resources required for its implementation within the institution. 

Bearing in mind the institutional purpose, nurses set the example and call for the student’s participation in a way that allows them to internalize the behaviour and skills required for their care and for others. Among their activities as *educators*, they offer health promotion and disease prevention programs according with the course of life, which include - among others, health education, intervention in the classroom, epidemiological surveillance, and vaccination according to the broadened immunization plan, information and communication related to health care, intervention in pedagogical projects and promotion of healthy lifestyles and of a culture that protects health. All these roles and responsibilities demand of nurses to *investigate* to update and share the professional and disciplinary knowledge, to strengthen the capacity of caring for the experience of the health of the members of the school community individually or collectively.

### Theoretical assumptions and their prioritizing

Table 1 shows the theoretical assumptions proposed in the study. In total, there were 36 prioritized assumptions classified into high, medium, or low levels. The assumptions of highest priority indicate which is most important for the school nurse and which has greatest control or governance, such as warm treatment, supported on the best evidence available and supported on adequate communication.

**Table 1:** Assumptions proposed as part of the development of the *Model of professional practice of school nursing for Colombia 2019*

Assumptions of school nursing	Score	Priority level
1) Kind treatment by nursing helps to establish a relationship of trust that favors caring for the members of the education community	500	High
2) To adequately care for a member of the school community, nurses must: assess and prioritize the care of their needs; identify the care actions required; establish with the person shared care goals; assist, support, or educate as required; and evaluate and register each of the actions undertaken	495	High
3) The members of the school community who receive warm and timely care by nursing feel satisfied and grateful	495	High
4) The care the nurse offers to the school community must be technically sound and based on the best evidence available	486	High
5) To provide safe care to students, adequate communication is necessary with their parents or guardians, as well as with the different areas of the school	482	High
6) A well-implemented nursing service generates trust in the students’ parents	477	High
7) Complete assessment that guarantees the information required and privacy necessary is indispensable to provide good nursing care	473	High
8) Education is a fundamental and multiplier tool for care that nursing has to promote the health of the school community and this must be adapted by taking into account the characteristics of the participants	468	Medium
9) School nurses must maintain continuous training to respond adequately to the health requirements of the education community.	468	Medium
10) To focalize the care required, the school nurse must understand that the child is in a period of growth and development, which generates changing needs	468	Medium
11) School nursing must be an integral part of the services of wellbeing offered by the school	468	Medium
12) School nurses must gather information from government entities related with health and transmit them in timely manner to the student community	468	Medium
13) Creativity and use of information and communication technologies provide school nurses a tool to support health care	468	Medium
14) In the school environment, nurses must ensure permanent protection of the children	464	Medium
15) The holistic care of the members of the school community must keep in mind the environment	459	Medium
16) The collective vision of health within the school community requires a program of epidemiological surveillance and corresponding actions of health promotion and disease prevention	459	Medium
17) To anticipate to requirements of care, in the school community, it is necessary to establish a support network for special situations and emergency cases	459	Medium
18) Nursing must support the institution to verify safe intra- and extra-curricular environments	459	Medium
19) The school environment is a privileged setting for nursing care that promotes health during the course of life	459	Medium
20) Caring for the education community with criteria of quality and opportunity demands having the resources necessary for said care	455	Medium
21) Improving the health care of the education community requires the co-responsibility from each of its members	450	Low
22) Nurses in the school environment are responsible for advising, supervising, and evaluating the auxiliary staff in their charge	450	Low
23) Nurses require developing management skills to suitably fulfill their activity of caring for the health of the education community	450	Low
24) Adequate care of the school community requires appropriate facilities to care for emergencies under current enabling regulations	445	Low
25) Nursing must have the human and physical resources necessary to safely care for the student population, besides covering the full school day, including extracurricular activities of academic, sports, and cultural nature	445	Low
26) Diminished scholar and labor absenteeism due to health motives is one of the goals of school nursing	445	Low
27) A school that prioritizes health care, reflects it through positioning nursing in its organizational scheme, its PEI, and its school life manual	445	Low
28) Nursing with adequate and pleasant areas generates in the students more pleasure to consult and seek help to care for their health	441	Low
29) Adequate nursing care demands registry and technological support for the school community	441	Low
30) The suitability of the school nurse permits academia to seek support on said nurse to broaden the educational offer	441	Low
31) To promote the safety of the school community, it is fitting to participate in identifying its risks and in planning and executing a program seeking to control and minimize them	432	Low
32) School nursing must stimulate the children’s development supporting their capacity to solve interpersonal problems not always related with health	427	Low
33) A nursing program in the school environment requires assignation of its own budget	414	Low
34) The nurse must participate in environmental health programs required by the school community	414	Low
35) Execution of an inclusive policy within the education community requires special actions with the respective resources	409	Low
36) The strategic location and quiet atmosphere of nursing support the wellbeing of the school community	405	Low

In synthesis, these concepts and prioritized assumptions comprised the initial version of the model of *professional practice of school nursing for Colombia*, which when validated by the participants received the slogan “*Education and health: a caring bond”.*

The model indicates that the practice of school nursing requires generating a caring bond, which unites education and health. For this, both the school environment and the kind treatment by the nurse are fundamental to establish a relationship of trust that favours caring for the members of the education community. The care nurses offer to the school community must be technically sound and based on the best evidence available. When caring, nurses apply and register the nursing process, in this, the full assessment, necessary privacy, and adequate communication with parents or guardians and with the different areas of the school, are necessary. 

A well-implemented nursing service generates trust in all the members of the school community, who upon receiving warm and timely care feel satisfied and grateful. During the external validation with academic experts, these classified the *Model of professional practice of school nursing for Colombia* by employing the criteria by Kim, based on which it was established that the model designed is of functional type, given that it reflects it directly a route to guide the practice of school nursing in the country; it is a model of humanistic orientation, given that it has the child or adolescent as the priority focus of action; it is of integral nature because it reflects a systemic vision of the nursing practice; it is representative because it portrays the reality lived by nursing at school community level; and - lastly - it was seen as a model, which allows monitor through the creation of indicators based on its assumptions.

## Discussion

The process of constructing and validating a model of professional practice of school nursing for Colombia generates a contribution to universal literature that reflects the search and support of theoretical models to guide the school nursing practice and its roles to guide the exercise of nurses in this field.(5) The result of present study complements prior research with similar purposes. In England, for example, through a qualitative study, a group of researchers sought to know what the perception of school nurses on their own role so that upon understanding it better, the practice would be qualified.(14) Although their findings indicate that the principal focus of school nursing is education for health, an activity that coincides with nursing roles described in this model, the authors also emphasize on institutional consultancy, workload, and search for educational resources and community work. 

An integrative review conducted in Brazil on health education given by nursing in schools, indicates - as in the present work - that this component of the school nurse’s role is fundamental. For them, this activity is organized in three areas that include: education for health generically; nursing and education on school health; and actions carried out in the school environment as part of the educational responsibility.(8) These three components are visible in the Colombian model of school nursing, however, their classification is complementary to such. 

Consideration and integration of the school community reflected by the present development was also studied in Brazil, where it was found that the work by these nurses with the families impacts positively on the better conduct of the students,(15) and in Norway, where it was evidenced that joint work between nursing and physical educators motivates the participation of children in activities that promote their health.(16) In the United States, to explain and guide nursing within the setting of school health, a group of nurses reported the adoption of the practice model *Intervention Wheel* with 17 possible public health interventions for people in individual, family(17), or community manner. Its use, although not dealing with a specific school nursing model, like the one developed in the present work, facilitated comprehension and permitted focalizing the public health component at school level. Likewise, in Australia, the presence of nurses to implement a structured health promotion program in the school reflected that it is possible to improve the health conducts of children and, thus, justified the funding required by these types of programs.(18) Similar findings are reported in another North American study, which indicates how the presence of full-time school nurses is necessary to diminish frequent diseases in children and adolescents and improve their academic performance.(19)

A systematic literature search on school nursing conducted in the United States grouped its activities into four: those of health promotion and prevention, those of categorization and treatment of acute health problems, management of children with chronic diseases or conditions, and psychosocial support to students; from its results, it is deducted that the presence of school nurses was associated with greater care, better school quality and higher savings, and it became evident that the members of the school’s education community, including teachers, school administrators, and parents, see the school nurse as a valuable member of the education team. However, the authors point out, that it is necessary to have greater evidence and methodological rigour for school nursing to be enhanced, increase its number of members, and achieve positioning their role, motive that supports a call to work in alliance between this group and academia from universities to strengthen this field.(20) The present development, which permitted obtaining a guiding model for school nursing in Colombia, is not only a reflection of the synergy generated by the joint work between school nurses and university professors, but which also reports to a broader nursing role, with a total and inclusive vision of students in their context. 

A work conducted in Australia indicates how nurses can impact the life projects of children, helping them to better understand their skills, to care for themselves and others.(21) As it occurs in the school-nursing model for Colombia, the centre of and focus of action is the wellbeing of students and their strengthening as protagonists of their history. Also contained in the present model is caring for children with diverse skills, which are supported to achieve their wellbeing and inclusion to school life. Works in the field reflect the role of the school nurse in facilitating this inclusion and call on strengthening the support that backs their actions, as well as that of other professionals,(22,23) explicitly, as done by the school-nursing model for Colombia indicating, as done in the present development, that for health promotion, student participation and satisfaction students with the programs are definitive.(24) Lastly, this conceptual progress agrees with current criteria internationally for the development of nursing theoretical models and which include internal and external criticism; theoretical representation and critical discussion; evaluation examination of nursing models and theories; and revision of formation of processes related with the practice.(25)

This study concludes that the *Model of professional practice of school nursing for Colombia: education and health: a care bond* identified the meta-paradigmatic concepts of the professional discipline in this field, as well as the assumptions relating them in the school practice to achieve the goals proposed. Validation of the theoretical development with the participating nurses ratified their identification with it. Adding to this, the contribution by experts in theoretical development of nursing and the comparison of the development achieved with international standards based on which the process and results obtained were ratified.

This model prioritizes the closeness of nurses to favour the care of the members in the education community. It demands technical soundness based on the best evidence available and guided by the steps of the nursing process, which will permit guiding the definition and evaluation of care goals, helping to maintain autonomy, as well as its interprofessional contribution to strengthening the care bond between health and education. 
